# Mechanistic insights into the anti-restenotic effects of HSP27 and HO1 modulated by reconstituted HDL on neointimal hyperplasia

**DOI:** 10.1038/s41598-023-49367-9

**Published:** 2023-12-12

**Authors:** Ye Ji Kim, Zinah Hilal Khaleel, Myeongji Jin, Jo Woon Yi Lee, Seongchan Park, Seongmin Ga, Nam Hyeong Kim, Deok Hyang Sa, Eun Sung Kang, Seul Hee Han, Ji Yeun Lee, Hyo Jung Ku, Sang-Wook Kim, Ki Yong Kim, Jeong Euy Park, Yong Ho Kim, Bok-Soo Lee

**Affiliations:** 1 SKKU Advanced Institute of Nanotechnology (SAINT), Sungkyunkwan University, 2066 Seobu-Ro, Suwon, 16419 Republic of Korea; 2 Department of Nano Science and Technology, Sungkyunkwan University, Suwon, 16419 Republic of Korea; 3 Division of Cardiology, Samsung Biomedical Research Institute, Sungkyunkwan University School of Medicine, Seoul, Republic of Korea; 4 Protein Research Lab, CRC, GC Biopharma R&D Center, Green Cross Co., Yongin, 16924 Republic of Korea; 5 Division of Cardiology, Samsung Medical Center, School of Medicine, Sungkyunkwan University, Seoul, 06351 Republic of Korea; 6 Department of Nano Engineering, Sungkyunkwan University, Suwon, 16419 Republic of Korea; 7 Department of Biomedical Engineering, Sungkyunkwan University, Suwon, 16419 Republic of Korea; 8 Center for Neuroscience Imaging Research (CNIR), Institute for Basic Science (IBS), Suwon, 16419 Republic of Korea; 9 Present Address: Samsung Bioepis PD Team, 76 Songdogyoyuk-Ro, Yeonsu-Gu, Incheon, 21987 Republic of Korea; 10 Present Address: Genexine, BioResearch Institute, 172 Magocjungang-Ro, BioInnovationPark, Bldg. Gangseo-Gu, Seoul, 07789 Republic of Korea

**Keywords:** Biological techniques, Biotechnology, Cell biology, Chemical biology, Molecular biology, Biomarkers, Cardiology, Molecular medicine, Pathogenesis

## Abstract

High-density lipoprotein (HDL) therapy has demonstrated beneficial effects in acute stroke and acute myocardial infarction models by reducing infarct size. In this study, we investigated the inhibitory effects of reconstituted HDL (rHDL) on neointimal hyperplasia and elucidated its underlying mechanism using a balloon injury rat model. Our finding revealed a significant 37% reduction in the intima to media ratio in the arteries treated with 80 mg/kg rHDL compared to those subjected to injury alone (*p* < 0.05), indicating a specific inhibition of neointimal hyperplasia. In vivo analysis further supported the positive effects of rHDL by demonstrating a reduction in smooth muscle cell (SMC) proliferation and an increase in endothelial cell (EC) proliferation. Additionally, rHDL treatment led to decreased infiltration of leukocytes and downregulated the expression of matrix metallopeptidase 9 (MMP9) in the neointimal area. Notably, rHDL administration resulted in decreased expression of VCAM1 and HIF1α, alongside increased expression of heme oxygenase 1 (HO1) and heat shock protein 27 (HSP27). Overexpression of HSP27 and HO1 effectively inhibited SMC proliferation. Moreover, rHDL-mediated suppression of injury-induced HIF1α coincided with upregulation of HSP27. Interestingly, HSP27 and HO1 had varying effects on the expression of chemokine receptors and rHDL did not exert significant effect on chemokine receptor expression in THP1 cells. These findings underscore the distinct roles of HSP27 and HO1 as potential regulatory factors in the progression of restenosis. Collectively, our study demonstrates that rHDL exerts a potent anti-neointimal hyperplasia effect by reducing leukocytes infiltration and SMC proliferation while promoting EC proliferation.

## Introduction

Restenosis is one of the major challenges in healthcare, occurring frequently after percutaneous coronary interventions (PCI). It is primarily attributed to neointimal hyperplasia, characterized by the hyperproliferation, migration, and accumulation of vascular smooth muscle cells (VSMCs). Stent-based approaches, coating stents with anticancer drugs such as rapamycin and paclitaxel, have been developed to efficiently inhibit cell proliferation^1,2^. Although drugs-coated stents adequately prevent intimal hyperplasia, they often result in incomplete reendothelialization and poor coverage of stent struts by smooth muscle cells, even long after stent implantation. Consequently, recent studies have focused on achieving controlled drug release directly into the artery using liposomes, micro- and nano emulsions, or bioresorbable stents. However, the occurrence of late stent thrombosis, potentially leading to fatal events, remains a serious concern^3,4^. Additionally, numerous experimental and clinical studies have demonstrated the crucial role of inflammatory responses in the pathogenesis of restenosis and neointimal hyperplasia^5,6^. Nevertheless, achieving effective cell growth regulation and anti-inflammatory effects continues to pose a significant challenge.

High-density lipoprotein (HDL) plays a crucial role in facilitating cholesterol efflux from macrophages, promoting its transport to the liver for excretion and reducing tissue deposits. In addition to its cholesterol transport function, HDL exhibits anti-inflammatory, anti-oxidative, and anti-thrombotic effects^7^. Vascular injury resulting from shear stress by the blood flow or oxidative stress triggers immune cell recruitment and activation, leading to inflammation^8^. Consequently, an optimal healing process following arterial wall injuries involves two essential components: rapid reendothelialization and inhibition of smooth muscle cell proliferation and migration. Restoration of the endothelial layer promptly and subsequent suppression of smooth muscle cell proliferation and migration are vital in the healing process of arterial wall injuries. HDL has demonstrated the ability to enhance reendothelialization and protect against endothelial cell death by mitigating oxidative stress^9,10^. These beneficial effects can be attributed to various components present within HDL particles, with particular emphasis on the quality of HDL^7,11^.

Oxidative stress-induced expression of hypoxia-inducible factor-1 alpha (HIF-1α) following vascular injury has been implicated in vessel sprouting and initial destabilization, contributing to vascular remodeling^12^. Disruption of the endothelial barrier and oxidative stress caused by reactive oxygen species (ROS) disturb intravascular homeostasis and have been associated in various diseases, including atherosclerosis, restenosis, and Alzheimer's disease^13^. Endothelial denudation leads to the upregulation of adhesion molecules and chemokines, triggering inflammation through leukocyte infiltration^14,15^. Heme oxygenase 1 (HO1), an inducible protein present in various cell types, has been shown protective effects against vascular constriction and proliferation by arresting the cell cycle during systemic inflammatory conditions and oxidant stress^16^. HDL has been found to inhibit vascular inflammation by inducing HO1^17^. Furthermore, HDL preserves the medial layer and attenuates atherogenesis, partially through upregulation of heat shock protein 27 (HSP27)^18^. Notably, altered expression of HSP27 has been observed in patients with acute coronary syndrome (ACS) and advanced atherosclerotic lesions^19^.

While small heat shock proteins (sHSPs) like HO1 and HSP27 are implicated in vessel homeostasis, the direct association between HDL's inhibition of neointimal hyperplasia and the functions of these proteins remains unclear. Thus, the objective of this study is to explore the mechanisms by which HDL modulates sHSPs in injured vessels, leading to a reduction in neointimal hyperplasia using a balloon injury rat model.

## Materials and methods

### Production of reconstituted HDL

HDL was prepared by mixing an apolipoprotein A-I (apoA-I) solution with a lipid solution containing soybean phosphatidylcholine (PC) and sodium cholate in a specific buffer. After diafiltration, sucrose was added and the lipoprotein solution was adjusted to a desired concentration before being sterile-filtered, lyophilized, and stored. Prior to infusion, the lyophilized rHDL was reconstituted with non-pyrogenic water for injection, resulting in disc-shaped particles resembling nascent HDL (“Supplementary Methods” in detail). ApoA-I was isolated from fraction IV paste after it underwent fractionation of human plasma by Green Cross Co., which was obtained from the Blood Center, Korea. The procedure strictly complied with the guidelines as set forth by the National Blood Management Act (Act No. 18626).

### Balloon injury rat model

Male Sprague-Dowley (SD) rats at 6 weeks of age were purchased from Charles River (Boston, MA) and housed in a temperature- and humidity-controlled animal facility in SBRI, SMC until weight reach 400–450 g. The rats were maintained under a 12 h light/dark cycle and had free access to standard food and water. To induce neointima formation, the right carotid artery of rats (n = 10) was exposed, inserted with a 2F Fogarty catheter in a defined arterial segment, then the endothelium was denuded by moving the catheter back and forth 3–4 times. At day 4 and 4 weeks, injured carotid arteries after perfusion with heparinized PBS followed by 10% formalin were isolated from rats and fixed in 10% neutral buffered formalin for 48 h. Fixed arteries were then embedded in paraffin, cut into 5 µM sections and were stained with H&E staining solution to assess neointima formation. All of our experimental protocols were approved by animal experimental committee at the Institutional Animal Care and Use Committee (IACUC) of the Samsung Biomedical Research Institute (SBRI), Samsung Medical Center (SMC). All animal experimental procedures were carried out in accordance with the ARRIVE guidelines.

### Histological examinations

The deparaffinized tissue sections were pretreated with hydrogen peroxide solution (DAKO, Denmark) for 30 min, washed once with a large volume of water and three times with PBS. Then the tissues were pretreated with antigen retrieval solution (DAKO, Denmark) and washed three times with PBS. After blocking with blocking buffer containing 10% BSA, tissue sections were incubated with antibodies against human apoA-1 (1:50 dilution), PCNA (1:200 dilution), CD68 (1:200 dilution), Matrix metalloproteinase 2 (MMP2) or 9 (MMP9) (Epitomics, Burlingame, CA), HIF 1a, and HO1 for 2 h to overnight at 4 ℃. Sections were washed with PBS three times and incubated with appropriate secondary antibodies conjugated with horse radish peroxidase (HRP) for 2 h. After washing with PBS, the tissue sections developed the positive signals by adding DAB solution (DAKO, Denmark) and counter-stained with hematoxylin. After mounting, images were visualized under *ECLIPSE 80i* light microscope (NIKON, Japan).

Colocalization of alpha smooth muscle cell (αSMC) actin (Abcam, Cambridge, UK) (Cell signaling, Danvers, MA) and PCNA or vWF and PCNA in the lesion were analyzed by confocal microscopy. To visualize the target proteins in the lesion, Alexa488-labeled antibody or Alexa 568 was used as secondary antibody (Molecular Probes, Paisley, UK) and observed at an excitation of 488 nm or 568 nm.

### Cell culture

Human aortic smooth muscle cells (HAoSMC) were purchased (C0075C, Gibco™, Thermo Fisher) and maintained with human VSMC basal medium (M231500, Gibco™, Thermo Fisher) with smooth muscle growth supplement (SMGS) (S00725, Gibco™, Thermo Fisher). Human aortic endothelial cells (HAoEC) were purchased (C12271, PromoCell, Heidelberg, Germany) and maintained with endothelial cell growth medium (C-22022 and C-39226, PromoCell). Human umbilical vein endothelial cell (HUVEC) was purchased (CC-2519, Lonza) and maintained with EGM™-2 Endothelial Cell Growth Medium-2 Bullet Kit (CC-3162, Lonza). THP1 was purchased (40202, Korean cell line bank) and maintained with RPMI 1640 medium (11875093, Gibco), 10% heat inactivated fetal bovine serum (FBS), penicillin (100 U/ml), and streptomycin (100 µg/ml) (Gibco, Carlsbad, CA). Cells were cultured at 37 ℃, at humidified 5% CO_2_ incubator unless mentioned otherwise. When reached confluence, the cells were sub-cultured using StemPro Accutase (A11105-01, Gibco). Culture media were changed every 3 days and passage numbers from 5 to 9 for primary cells were used for the experiments.

### Cell Proliferation Assay

Cell proliferation was determined using cell counting kit-8 (CCK-8) (Dojindo, Rockville, MD). The quintupled cells per condition were serum-starved for 24 h in 96 well plate. After media were replaced with complete media, the cells were then incubated for 1 h with or without rHDL (100 µg/ml) and the cells were cultured for further 48 h in stimulating with or without 10 ng/ml of VEGF for endothelial cells or 10 ng/ml of PDGF for smooth muscle cells. The cells were washed once with fresh media, added with 10 µl CCK-8 dye and incubated for 1 h at 5% CO_2_ incubator. The cell proliferation was calculated by measuring absorbance of colored product at 450 nm using microplate reader (BIO-RAD, Hercules, CA).

### HSPs K/D, overexpression of HSP27, and RT-PCR

To knock-down the HSPs, scrambled siRNA, siHSP27 or siHO1 were prepared by custom order from Bioneer. The siRNA sequences were as follows: siHSP27, siHO1, and scrambled siRNA (Supplementary Table 1). THP1 cells were then transduced 250 nM of siRNAs using RNAiMax (Invitrogen) for 6 h following wash once with fresh media, the cells were treated with or without 100 µM rHDL or 100 µM oxLDL for 18 h.

To assess the direct impact of HSPs on SMC proliferation, scrambled or siHSP27 was transfected into HAoSMC, which were then plated and cultured overnight in a 96-well plate. The cells were subsequently infected with a virus at a multiplicity of infection (MOI) of 10, obtained from an adenoviral vector containing HSP27, for 6 h. After a single wash with media, the cells were treated with either 100 µg/ml of rHDL or PBS for 42 h. Proliferation was then assessed using a CCK-8 assay kit.

Total cellular RNA was extracted using the Trizol reagent (Invitrogen) or RNA extraction kit (Invitrogen) according to the manufacturer’s instructions. For cDNA synthesis, 1 μg of total RNA was reverse transcribed using cDNA synthesis kit (Bioneer, Daejeon, Korea) at 42 °C for 1 h. The PCR were performed 35 cycles using PCR amplification kit (Bioneer). After initial predenaturation step at 94 °C for 2 min, cDNA was amplified for 35 cycles with 10 pm of following primers: GAPDH, HSP27, HO1, CCR2, CCR5, CCR7, VCAM1, and, CX3CR1 Supplementary Table 2).

The amplification products were resolved by electrophoresis on 1% agarose gel with DNA staining dye. The images were captured by ChemiDoc (BioRad) and quantified by image J.

### Statistical analysis

Statistical differences were analyzed with unpaired Student’s *t* test or ANOVA with recommended correction using PRISM 10.0.1. Results are expressed as a mean ± SD.

## Results

### rHDL inhibits neointimal hyperplasia in balloon injured rat carotid arteries

**Figure 1:**
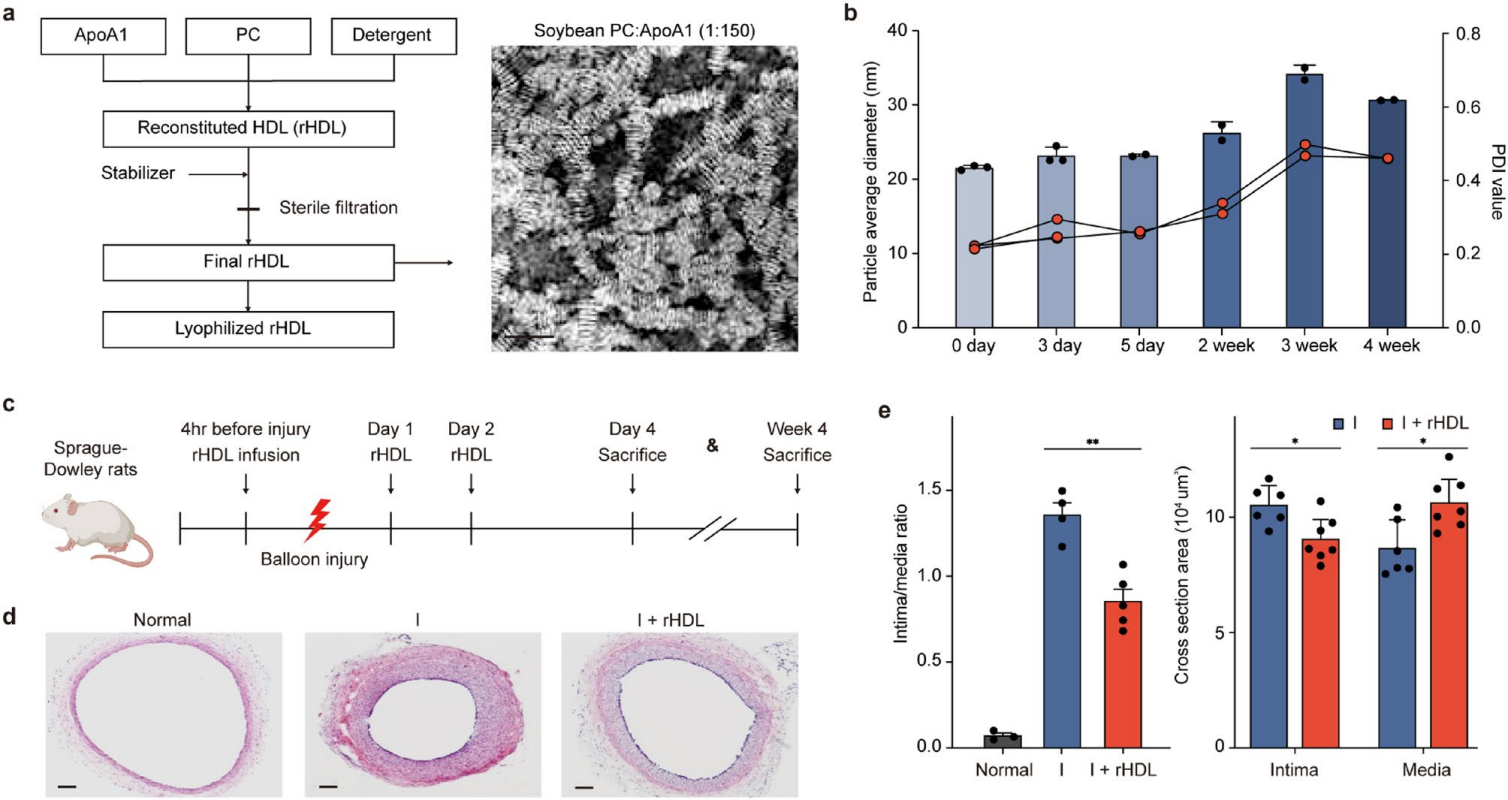
rHDL shows effects on neointimal hyperplasia in balloon injured rat carotid artery. (**a**) Procedure for reconstituting HDL and its structural analysis. (**b**) The stability of rHDL was assessed by measuring the particle average diameter and polydispersity index value. (n = 2–3). (**c**) Mice were divided into two groups: one group received intravenous administration of rHDL (80 mg/kg/day) (Group A, n = 13), while the other group received saline (Group B, n = 10). Administration occurred 4 h prior to balloon injury, as well as one, two and four days after balloon injury. (**d**) Neointima formation was visualized in representative cross-sections of the common carotid artery using H&E staining (magnification X100). Scale bar, 100 μm. (**e**) The extent of restenosis was evaluated by calculating the intima to media ratio (left) (**p < 0.01 versus injury group, Student t test, n = 4–5) and by measuring the areas of neointima and media (right) (*p < 0.05 verse injury group, Student t test, n = 6–7). *I* injury, *I + rHDL* injury model with rHDL infusion.

rHDL, generated according to the methods outlined, exhibited a discernible discoidal structure as observed in the electron microscopy image (Fig. 1a). This structural integrity persisted for up to 2 weeks, with an average particle diameter of 23.321 nm and an average PDI value of 0.255 (Fig. 1b). In our experimental model using Sprague-Dawley rats, an infusion of 80 mg/kg of rHDL was administered both prior to and after balloon injury (Fig. 1c). Notably, the administration of rHDL effectively suppressed the formation of neointima compared to the injury only group (Fig. 1d). The treated rHDL group exhibited a remarkable 37% reduction in the intima to media ratio compared to the injury group, accompanied by significant alterations in the area of both the intima (*p* < 0.05) and the media (*p* < 0.01) (Fig. 1e).

### rHDL modulates proliferation of vascular endothelial and smooth muscle cells

**Figure 2:**
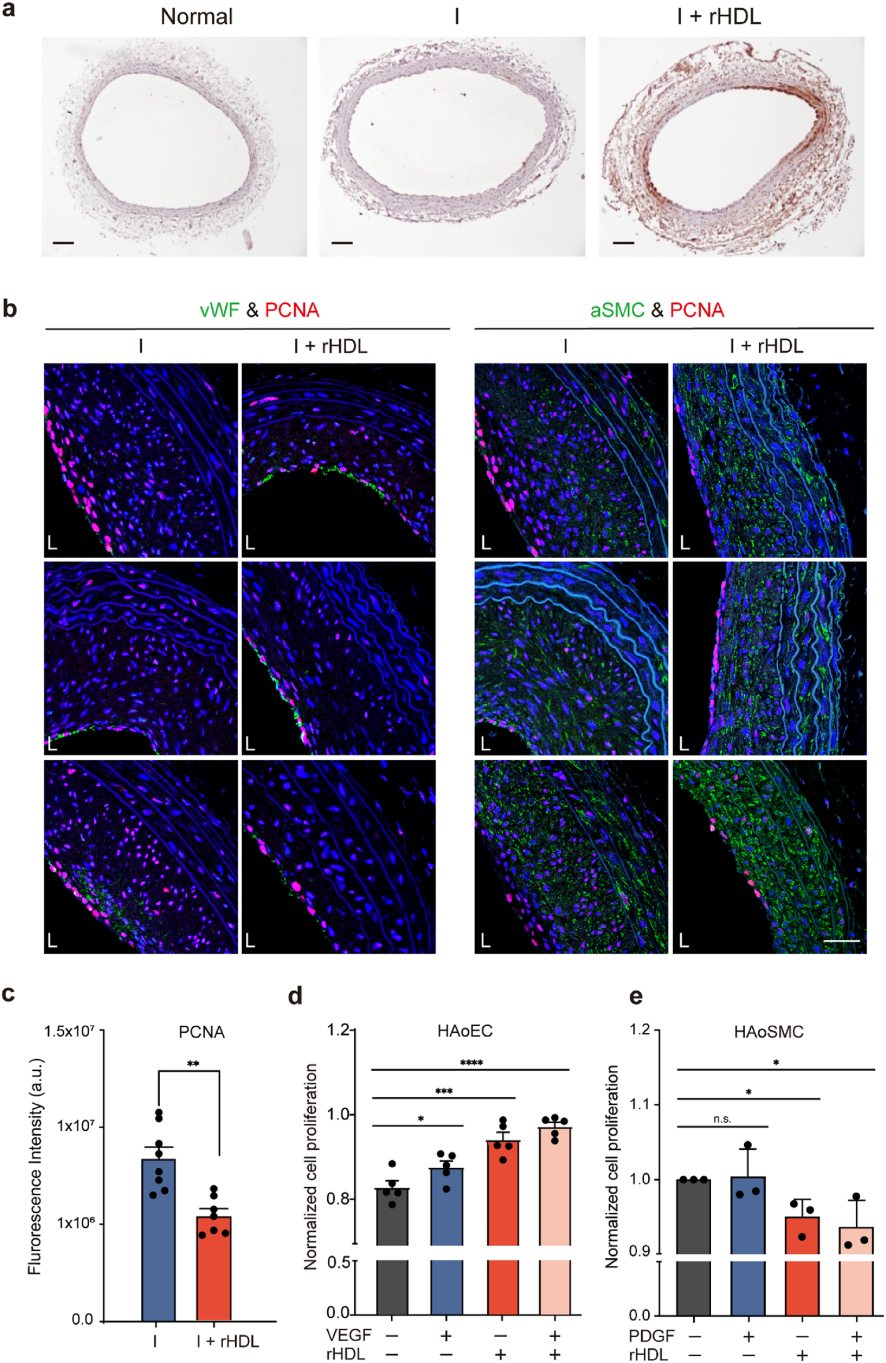
rHDL decreases cell proliferation in balloon-injured carotid arteries and differentially modulates proliferation of endothelial cell and smooth muscle cell. (**a**) Immunohistochemistry was performed on tissue sections of balloon injured rat carotid arteries at day 4 using an anti-human ApoA-I antibody to detect the presence of administered rHDL. Scale bar, 100 μm. (**b**) Double staining with anti-vWF (green) or anti-αSMC (green) and anti-PCNA antibodies (red), followed by DAPI staining (blue), was conducted on tissue sections of balloon injured rat carotid arteries from the injury group with or without rHDL treatment. Confocal microscopy (CLSM700, Leica, Germany) was used to capture the images and three images from both were shown. Scale bar, 20 μm. (**c**) Signal intensity of PCNA was analyzed from the images of (**b**). (Two-tailed paired t-test, *p < 0.05). (**d**) Quintupled endothelial cells (HAoECs) and triplicated HUVEC or human aortic smooth muscle cells (HAoSMCs) were cultured for 48 h in the presence or absence of different concentrations of rHDL. Cell proliferation was assessed using the CCK-8 assay kit by measuring absorbance at 450 nm after 1 h incubation (ns; non-significant, *p < 0.05, **p < 0.01, and ***p < 0.001, ****p < 0.0001 verse control group, one-way ANOVA, n = 3–5).

Accumulation of rHDL was visualized through ApoA-1 staining, confirming its presence across the tissue (Fig. 2a). Neointimal hyperplasia, characterized by the proliferation of endothelial cells and SMCs, was assessed to evaluate the effects of rHDL in an in vivo by co-staining with antibodies against cell specific markers and PCNA, cell proliferation marker. At 4 weeks post-balloon injury, the recovery of the endothelium, as indicated by vWF-positive areas, remained weak but co-localized with PCNA signal in rHDL treated group (Fig. 2b, left). Although PCNA signal did not overlay with SMC-positive signal, but it was dispersed throughout the whole area of tissue sections (Fig. 2b, right). Interestingly, the administration of rHDL seemed to augment vWF signal in 4 days compared with injury group while SMC signal did not show any significant differences (Supplementary Fig. 1). Proliferating signals in endothelial cells were predominantly observed 4 days after balloon injury in the rHDL-treated group. In contrast, the proliferation signal in SMCs was predominantly observed in the injury group at 4 weeks of injury and significantly decreased in the rHDL treated group (Fig. 2c). As supplement IHC data also indicate that two independent proliferating markers, PCNA and Ki-67, clearly showed rHDL inhibits cell proliferation in neointima layer (Supplementary Fig. 2).

The effect of rHDL on cell proliferation was confirmed in HAoECs (Fig. 2d), HUVEC (Suppl Fig. 3), and HAoSMC (Fig. 2d) by in vitro analysis using a CCK-8 dye-based assay kit. The data demonstrated that rHDL significantly enhanced endothelial cell proliferation (*p* < 0.0001) while reducing the proliferation of SMCs (*p* < 0.05) (Fig. 2c). These findings conclude that infusion of rHDL facilitated re-endothelialization and decreased the proliferation of SMCs.

### rHDL attenuates leukocyte infiltration and inhibits adhesion molecule expression as an anti-inflammatory response

**Figure 3:**
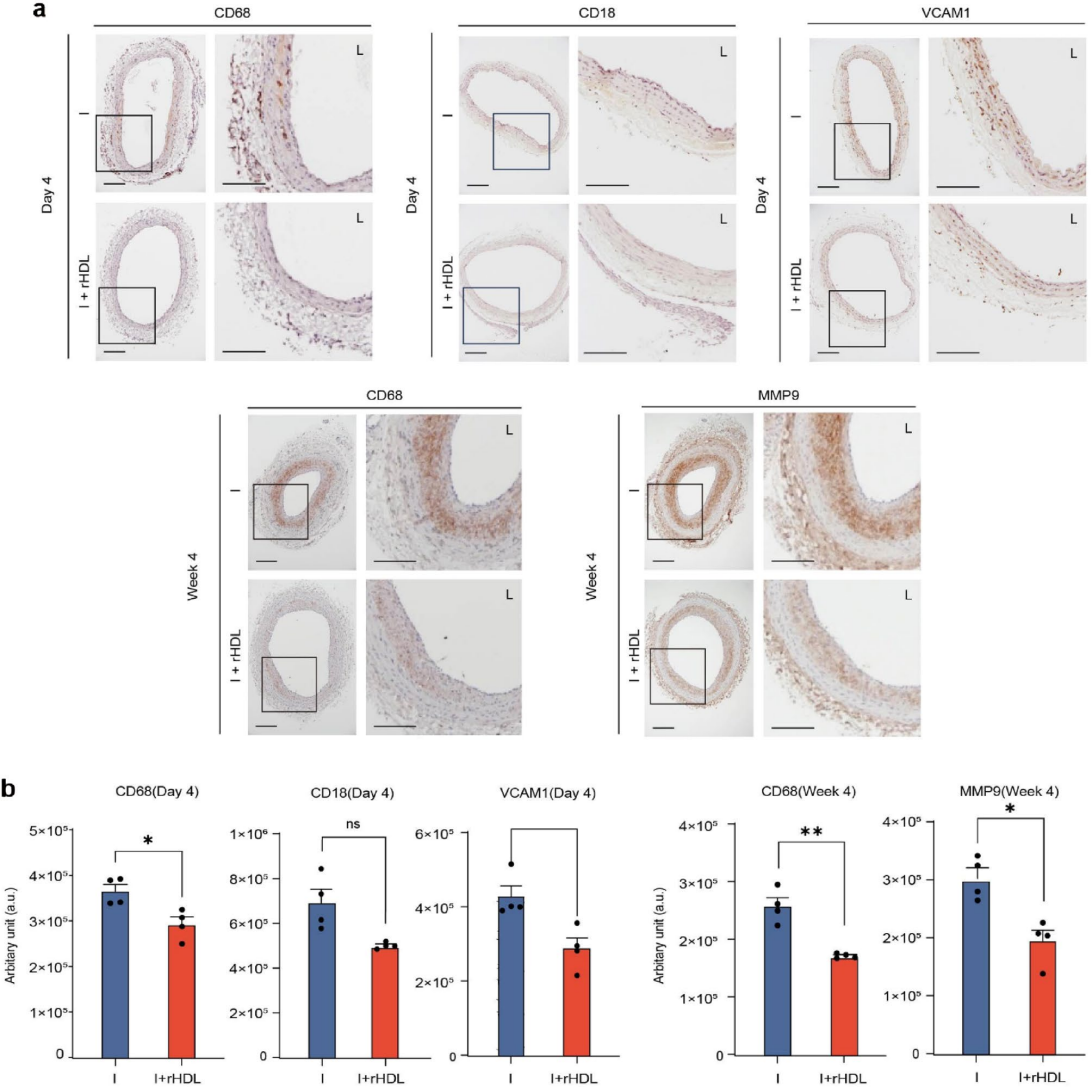
Anti-inflammatory effects of rHDL are associated with prevention of neutrophil/monocyte infiltration and binding with adhesion molecules. (**a**) Top. Immunohistochemistry (IHC) of CD18, CD68, and VCAM1 in tissue sections of balloon injured rat carotid arteries on day 4. Scale bar, 100 μm. (**a**) Bottom. Immunohistochemistry of CD68 and MMP9 in tissue sections of balloon injured rat carotid arteries on week 4. (**b**) IHC images were quantified by image J software. The intensities were showed as arbitrary unit (a.u.) and statistically analyzed between the two groups by paired t-test.

Inflammation can be triggered by the infiltration of leukocytes, which bind to adhesion molecules on damaged endothelial cells, leading to the differentiation of monocyte into macrophages. To investigate the effects of rHDL, markers of neutrophils (CD18), monocytes (CD68), and vascular cell adhesion molecule 1 (VCAM1) were examined. At day 4 after injury, representing the early stage of inflammation, CD18, CD68, and VCAM1 were detected in the media layer, while their detection was reduced with rHDL treatment (Fig. 3a). The presence of inflammatory cells, such as monocytes and other leukocytes, in the adventitia of blood vessel is a noteworthy observation. These suggest that immune cells may have originating from a source external to the blood vessel, indicating a potential connection between the adventitia and the recruitment of these cells. The efficacy of rHDL in preventing neointimal hyperplasia becomes apparent and observable by week 4. In tissue sections obtained at both the day 4 and week 4, there was a notable and statistically significant reduction in the CD68 signal, a marker indicative of residual macrophage, in response to rHDL treatment. These decrease in CD68 signal was concomitant with a marked reduction in the MMP9 secretion (Fig. 3b). These findings indicate rHDL efficiently suppresses inflammatory response in injured lesions.

### HSP27 and HO1 partly regulate the expression of chemokine receptors

**Figure 4:**
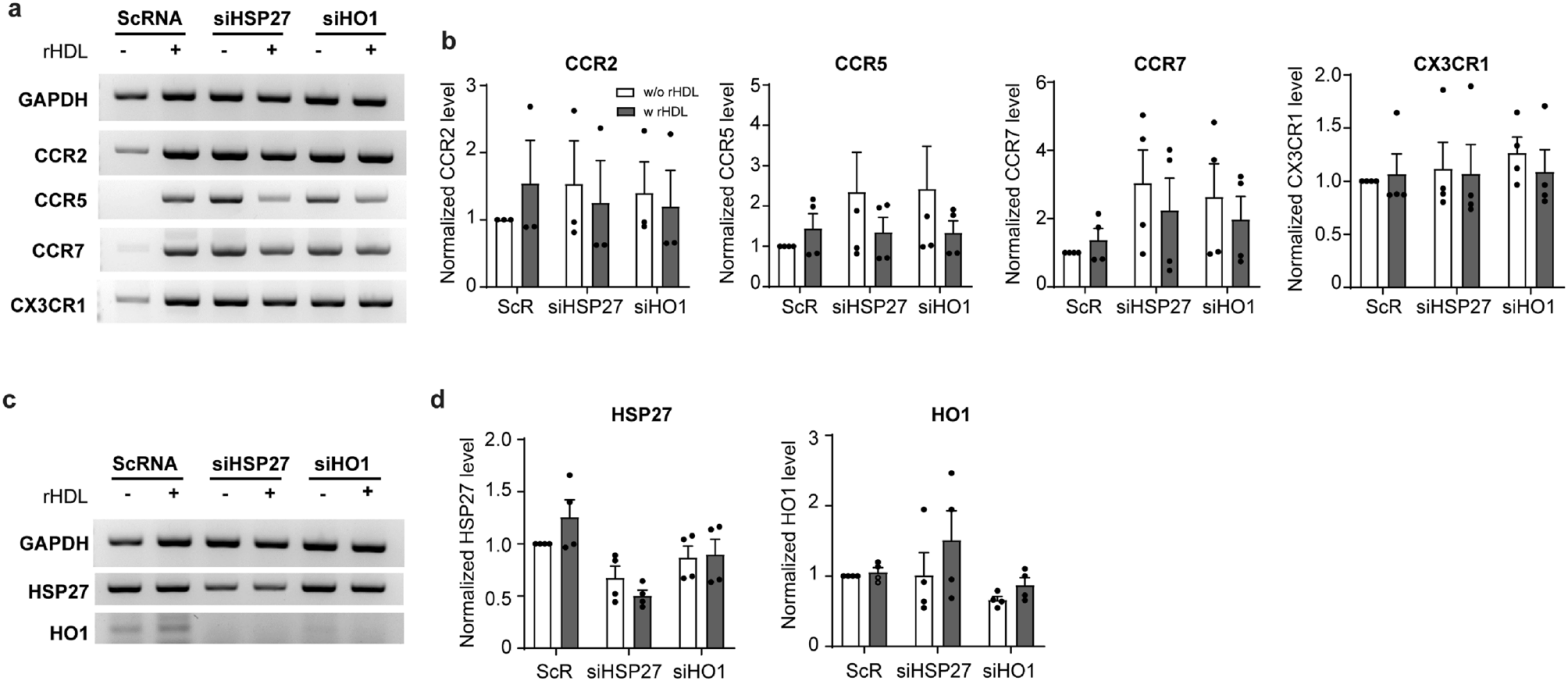
Regulation of expressing chemokine receptors under knockdown of HSP27 and HO1. (**a**) Gel-based RT-PCR analysis of chemokine receptors, CCR2 CCR5, CCR7, and CX3CR1 with HSP27 or HO1 knock downed, respectively, in THP1 cells. (**p < 0.01, ***p < 0.001, ****p < 0.0001 verse scrambled siRNA, one-way ANOVA, n = 2–3). (**b**) Quantifying the expression levels for CCR2, CCR5, CCR7, and CX3CR1 are analyzed by image J software, respectively. (**c**) Gel-based RT-PCR analysis of HSP27 and HO1 expression in HSP27 and HO1 knock downed THP1 cells. (**d**) Knocked down levels are quantified for HSP27 and HO1, respectively. (ns; no significant. *p < 0.05, **p < 0.01 verse scrambled siRNA, one-way ANOVA, n = 2–3).

C–C motif chemokine receptors (CCR) and CX3C motif chemokine receptors 1 (CX3CR1) are involved in infiltration of leukocyte and inducing inflammatory responses. To investigate the involvement of HSP27 and HO1, THP1 cells were introduced with siRNA to knockdown of HSPs and check the expression level of chemokine receptors (Fig. 4a,b). HSP27 and HO1 knockdown increased the expression of CCR2, CCR5, and CCR7 but it was not statistically significant. Notably, CX3CR1 expression did not affect by HSPs knock down (Fig. 4a,b). Although oxLDL is well known factor to decrease CCR expression in atherosclerotic condition, the direct role of rHDL on CCR expression was not known. In contrast to oxLDL, rHDL did not exert a significant impact on HSPs-mediated CCR inhibition (Fig. 4a). The original, uncropped images are provided in Supplementary Fig. 4. These finding suggest that rHDL may partly suppresses CCR expression through HSPs. The suppressive role could be accomplished through an alternative pathway distinct from the involvement of HSP27 or HO1. The efficiency of knock-down of HSP27 and HO1 is shown in Fig. 4c,d. Since the role of HSP27 is involved in phosphorylation than quantity, function of HSP27 were tested in cells overexpressing wild-type (wt) or mutated HSP27 as described in Supplementary Fig. 5. It is worth notifying that while the expression of other tested chemokines remained unaffected, IL8 expression was inhibited in cells overexpressing wtHSP27. However, the intricate mechanism underlying this specific regulation require further in-depth investigation to clarify.

### rHDL directly regulates expression levels of HSP27 to inhibit SMC proliferation

**Figure 5:**
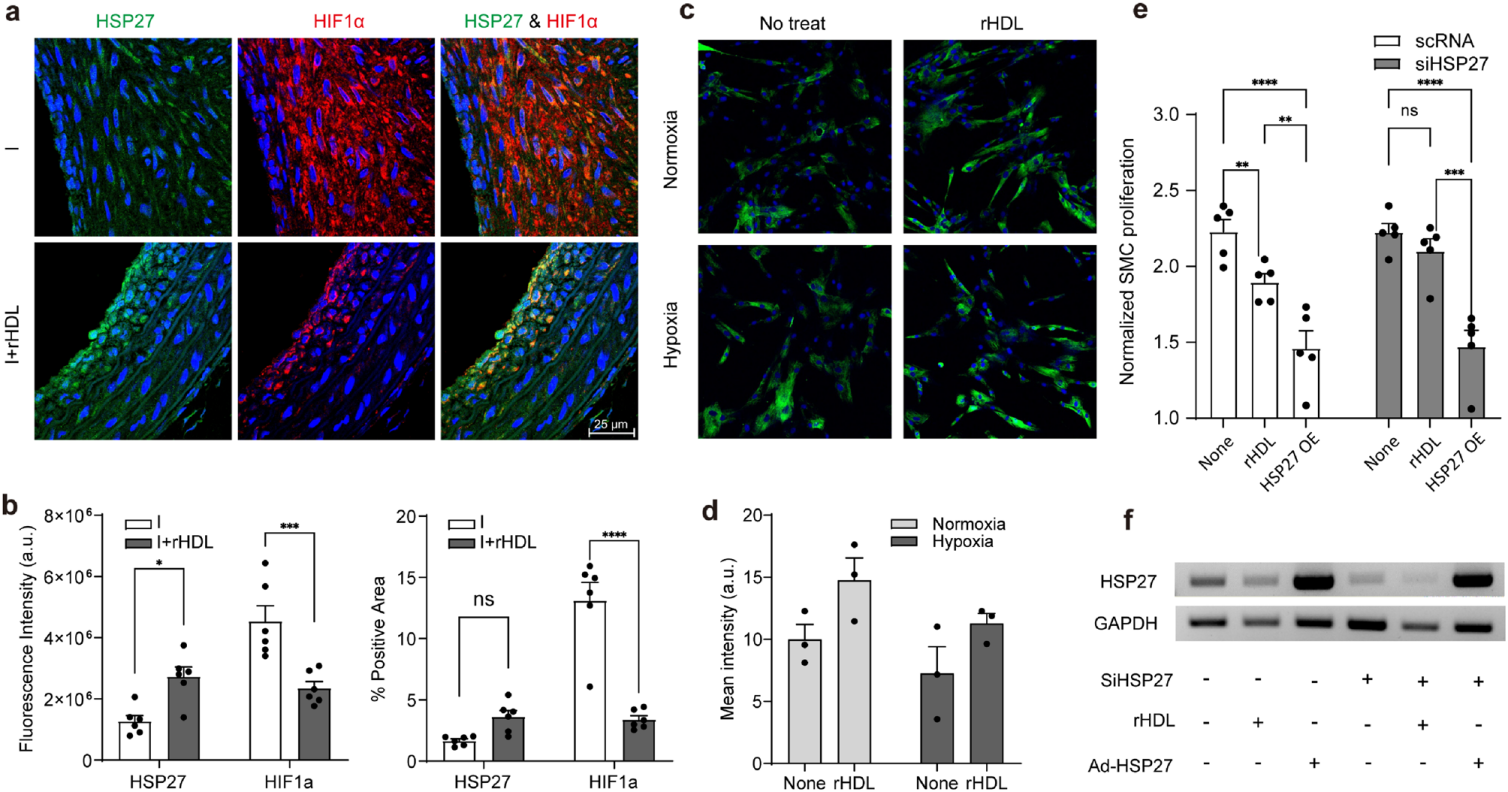
rHDL-mediated SMC proliferation via HSP27. (**a**) The expression level of HSP27 and HIF1α in tissue sections from balloon injured rat carotid arteries on week 4 with or without rHDL infusion was analyzed by confocal analysis. The one representative image is shown. (**b**) The fluorescence intensities were measured using image J software. The graph represented as arbitrary unit (a.u.) after normalizing by vessel area (**b**. left) or % signal positive area (**b**. right) (**p < 0.01 verse injury group, one-way ANOVA, n = 3). (**c**) HSP27 induction by rHDL were analyzed both on normoxia (left) or hypoxia (right) conditions in cultured SMCs and the expression level was quantified by image J analysis followed by Student t-test (**d**). (**e**) Loss- or gain of function for HSP27 on SMC proliferation were examined after KD HSP27 followed by with or without HSP27 overexpression by Ad-HSP27 infection, then CCK-8 assay as described in Materials and Methods section. Statistics were analyzed by two-way ANOVA with Turkey’s multiple comparisons (ns; no significant. *p < 0.05, **p < 0.01 and ***p < 0.001). The level of HSP27, HO1, and GAPDH in the experimental conditions was confirmed by RT-PCR (**f**).

HSPs, particularly HSP27 and HO1, play a critical role in promoting cell survival under conditions of damage, stress, or other forms of stimulation. To investigate the impact of rHDL on neointimal hyperplasia in balloon-injured rat carotids, the expression levels of HSP27 and hypoxia inducible factor alpha (HIF1α) was analyzed through confocal microscopy. As demonstrated in Supplementary Fig. 6a, HIF1α expression is most pronounced on day 4 following balloon injury and the infusion of rHDL markedly suppresses this induction of HIF1α. Even though the HIF1α signal is less intense during week 4 after injury, rHDL infusion continues to significantly reduce the signal, which is accompanied by a decrease in neointimal hyperplasia. As depicted in Fig. 5a, the infusion of rHDL led to a statistically significant increase in HSP27 expression, while concurrently, the signal for HIF1α was significantly reduced in comparison to the injured group (Fig. 5a,b and Supplementary Fig. 6b).

To clarify the role of HSP27 by rHDL, we conducted an in vitro study to investigate the loss or gain of HSP27 function. HAoSMCs demonstrated a mild induction by 100 μg/ml rHDL under both normoxic and hypoxic conditions (Fig. 5c,d). To assess whether HSP27 directly affects cell proliferation, we initially transduced HAoSMCs with siHSP27, followed by infection with an adenovirus containing HSP27 to rescue the knockdown condition. Subsequently, the cells were cultured in the presence or absence of rHDL. Notably, SMC proliferation was substantially inhibited in cells overexpressing HSP27, as depicted in Fig. 5e. Conversely, in rHDL-treated cells, there was a modest inhibition of SMC growth, which corresponded to a slight increase in HSP27 expression, as indicated by normalized expression levels (Fig. 5f). The original, uncropped images are provided in Supplementary Fig. 7.

These in vivo and in vitro data strongly suggest that rHDL plays a critical role in preventing neointimal hyperplasia by modulating the expression of HSP27 and HIF1α.

### rHDL regulates cell proliferation through modulation of HO1 expression

**Figure 6:**
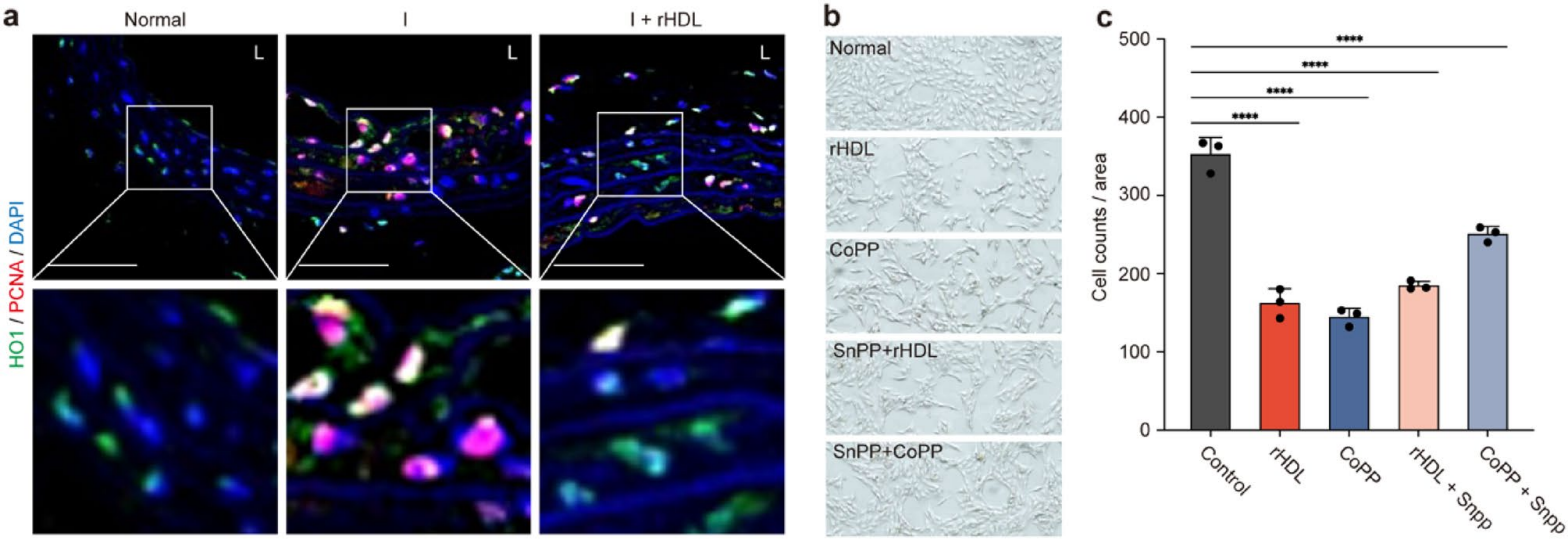
Inhibitory effect of HO1 on rHDL-mediated SMC proliferation in vivo and in vitro (**a**) Immunofluorescence staining of tissue sections from balloon injured rat carotid arteries with or without rHDL treatment. The sections were co-stained with anti-HO1 antibody (green), anti-PCNA antibody (red), and DAPI (blue). Confocal microscopy was used to capture the images (Magnification X400) Scale bar, 20 μm. (**b**) Rat vascular smooth muscle cells (RVSMC) were cultured for 48 h in the presence or absence of rHDL or cobalt protoporphyrin (CoPP) with or without tin protoporphyrin (SnPP). After fixation, cells were visualized using light microscopy (Olympus, Magnification X100). (**c**) The cell count and area calculation were performed using Image J software. (****p < 0.0001 verse Control, one-way ANOVA, n = 3).

To assess the direct impact of HO1 on neointimal hyperplasia, tissue sections were immunostained with a cell proliferation marker (PCNA) and HO1. The results provided clear evidence that cells in tissue sections with lower or no HO1 expression clearly exhibited a stronger PCNA signal, whereas cells with HO1 overexpression exhibited weak or no signal of PCNA (Fig. 6a). As depicted in Fig. 6b,c, treatment with rHDL effectively suppressed in vitro cell proliferation. However, this inhibitory effect was slightly attenuated when tin protoporphyrin (SnPP), an inhibitor of HO1 induction, was present. Notably, treatment with cobalt protoporphyrin (CoPP), which is known to generate carbon monoxide (CO), significantly inhibited cell proliferation, and this inhibitory effect was reversed when SnPP was co-administered. The original, uncropped images are provided in Supplementary Fig. 8. These findings indicate that rHDL may play a significant role in suppressing SMC proliferation through rHDL-mediated HO1 induction via the effector molecule CO.

## Discussion

Many trials have been conducted to address the issue of restenosis following in-stent surgery in patients with coronary artery disease. Drug-eluting stents (DES) coated with rapamycin or paclitaxel, are mostly well-known anti-restenotic approaches, however, those have been associated with undesirable side effects, including incomplete coverage of stent struts, fibrin deposition, and thrombosis after stenting^2,20,21^. Given the positive results of our previous study, which demonstrated that rHDL produced by Green Cross Corp. can prevent atherosclerotic progression in lesions in hyperlipidemic apoE−/− mice^18^, we sought to investigate whether rHDL could also prevent neointimal hyperplasia using a balloon injury rat model.

In this study, we observed that three administrations of rHDL significantly inhibited the development of neointimal hyperplasia, leading to a remarkable 37% of decrease in the intima-to-media (I/M) ratio (*p* < 0.001) by the 4th week. This effect was achieved by reducing the size of the neointima while preventing the integrity of the media layer (Fig. 1e, right). Previous research by Vanags et al. demonstrated that Apo A-I improves biocompatibility of stents by suppressing key inflammatory mechanism that initiate in-stent neoatheroscelrosis and enhance the removal of cholesterol efflux from macrophages within atherosclerotic plaque, using a mouse carotid-interposition graft in-stent model^22^. Their findings highlighted the role of Apo A-I in reducing platelet activation, macrophage infiltration, and promoting the re-growth of aortic endothelial cells over the stent strut. Likewise, in our study, we observed that rHDL significantly increased the proliferation of HUVEC and HAoEC proliferation (Fig. 2d and Supplementary Fig. 3), while decreased HAoSMC proliferation (Fig. 2e). These results were further supported by clear evidence of in vivo re-endothelialization, as indicated by a strong co-localization signal of vWF and PCNA in vessels infused with rHDL (Fig. 2b, left). In the same vessels, co-localization signal of SMC and PCNA clearly showed that rHDL inhibit SMC proliferation (Fig. 2b, right, Fig. 2e, Supplementary Fig. 2). In addition, rHDL reduced the expression of integrin accompanied with a reduction of MMP9 expression in injured vessels (Fig. 3), suggesting improvement in the stability of the SMC layer. To confirm the presence of human Apo A-I accumulation in rHDL infused carotid arteries, we examined the effect of rHDL on endothelial cells and SMC proliferation in vitro and in vivo (Fig. 2).

While studies have attempted to shed light on vascular injury and neointimal proliferation, the precise relationship between these phenomena remains elusive. Some investigations have proposed that neointimal hyperplasia is triggered by progenitor cells originating from the bloodstream, while others have indicated that progenitor cells stemming from the adventitia or endothelial progenitor cells within the injured endothelium also contribute to this process^23,26^. Furthermore, certain studies have reported that HDL or Apo A-I can expedite rapid re-endothelialization by promoting the recruitment of endothelial progenitor cells within the endothelium. These cells subsequently serve as a source of SMCs that proliferate at the injured sites^3,23^. Notably, scavenger receptor-BI is closely linked to the role of rHDL^10,24^. In a study by Deiner et al., it was elucidated how the transfer of the VEGF gene into the adventitial region effectively prevent lumen loss by promoting favorable arterial remodeling following PTCA in porcine coronary arteries^25^. In consistent with studies that vessel formation initiates from the adventitia^26,27^, we also found that rHDL significantly inhibits the infiltration of inflammatory cells into the neointimal area, particularly during the early stages, originating from the adventitia (Fig. 3). These findings strongly suggest that rHDL holds promise as a therapeutic candidate for preventing neointimal hyperplasia.

To explore the effects of HSP27 and HO1 on the infiltration of blood leucocytes to injured vessel, we conducted knockdown experiments targeting both of these HSPs. Prior studies have established that specific chemokine receptors, such as CCR2, CCR5, and CX3CR1, play distinct roles in the development of atherosclerotic plaque^28^. CCR2 and CCR5 are essential for cell trafficking, while CX3CR1 is induced in macrophages to facilitate adhesion to SMCs^29^. Interestingly, rHDL has been found to promote plaque regression by enhancing the transformation of macrophages into the M2 state, which is associated with increased CCR7 expression^30^. Importantly, when we knocked down HSP27 or HO1, it differentially regulated the expression of chemokine receptors, including CCR2, CCR5, CCR7, and CX3CR1. In addition, rHDL did not exert a profound inhibitory effect on the expression of chemokine receptors. It is worth noting that under inflammatory conditions, results may differ. Nonetheless, these findings suggest that both HSP27 and HO1 may contribute, at least in part, to the processes of monocyte infiltration, accumulation, and egression (Fig. 4a,b). An intriguing observation was that knocking down HO1 reduced HSP27 expression, while knocking down HSP27 did not affect HO1 expression, suggesting that HO1 and its product, CO, can act as effector molecule of HO1 (Figs. 4 and 6).

In the context of inflammation, certain cytokines, such as CCL2, CCL5, and CX3CL1, stimulate SMC proliferation. It’s worth mentioning that HDL exhibits strong anti-inflammatory and anti-proliferative functions in SMCs when exposed to TNFα. This occurs through the inhibition of NF-κB and AKT, and ERK activity^31^. In line with this research, our findings suggest that rHDL not only modulates the expression of chemokine receptors via HSPs in monocytes but also reduces the expression of chemokines in SMCs. This dual actions on inflammation among immune cells and the proliferation of SMCs at injured sites may be more effective in controlling restenosis, while exerting a milder influence on cell function. Notably, HSP27 is known to be secreted into bloodstream, and its role in the extracellular space has been studied. It can activate NF-κB in macrophages, leading to the induction of both anti-inflammatory and pro-inflammatory cytokines, which can impact immune cell infiltration and eggression^32^. Additionally, HSP27 functions as a chaperone, with its roles varying based on its phosphorylation status and oligomer pattern^33^. An intriguing observation was made when we introduced an adenoviral vector containing wild-type HSP27 or a mutant HSP27 with three Ser residues mutated to Ala at well-known phosphorylation sites. Notably, the mutant HSP27 failed to inhibit IL8 expression in THP1 cells treated with oxidized LDL (Supplementary Fig. 5), indicating the need for further research to precisely understand the role of HSP27 in restenosis and atherosclerosis.

Oxidative stress often contributes to the deterioration of injured lesions^7,34^. Therefore, we investigated how rHDL influences the regulation of HIF1α in our animal model. In a previous study, we demonstrated that neointimal hyperplasia was inhibited by treatment of herbal extract called HMC05, via HSP27 over-expression^35^. In our current study, we observed the induction of HSP27 and the reduction of HIF1α in vivo following rHDL infusion (Fig. 5a,b). This reflects that rHDL-mediated induction of HSP27 seen in cultured HAoSMCs (Fig. 5c,d). Notably, when we overexpressed HSP27 in HAoSMCs through adenoviral vector gene delivery in cells where HSP27 was knocked-down HSP27 (Fig. 5e), we observed a significant reduction in SMC proliferation. Moreover, the relatively modest induction of HSP27 by rHDL (Fig. 5e,f) resulted in a more moderate inhibition of SMC proliferation. However, when we knocked down the genes, the inhibitory function was not significantly restored (Fig. 5e,f). These results likely suggest that a complete knockdown of the target gene is required to observe the loss of function (Supplementary Fig. 7). These findings imply that the expression of adhesion molecules or cytokines is differentially influenced by the quantity or phosphorylation status of HSP27 and/or HO1. A study by Duckers et al. demonstrated that HO-1 inhibits VSMC proliferation in vascular injury model in pigs via modulation of cell cycle progression regulatory proteins, such as p21cip1 and p27kip1^16^. Another study revealed that the HO1/CO system plays an inhibitory role in atherosclerotic plaque formation, independently of its regulation of serum lipids or ox-LDL levels in rabbit model^36^. Importantly, we found that treating HAoSMCs with cobalt protoporphyrin (CoPP) or rHDL resulted in the inhibition of cell proliferation, and this inhibitory effect was reversed by SnPP treatment (Fig. 6). These findings suggest that HO1 might be a key player and that CO acts as the effector molecule in the rHDL-mediated inhibition of neointimal hyperplasia. Our previous results indicated that the overexpression of HIF1α in injury group was significantly reduced in animals treated with rHDL, not only in early stage (Supplementary Fig. 6) but also in later stage (Fig. 5a,b). Altogether, we can suggest that HSP27 and HO1 via HIF1α may play equally important inhibitory role in SMC proliferation.

**Figure 7:**
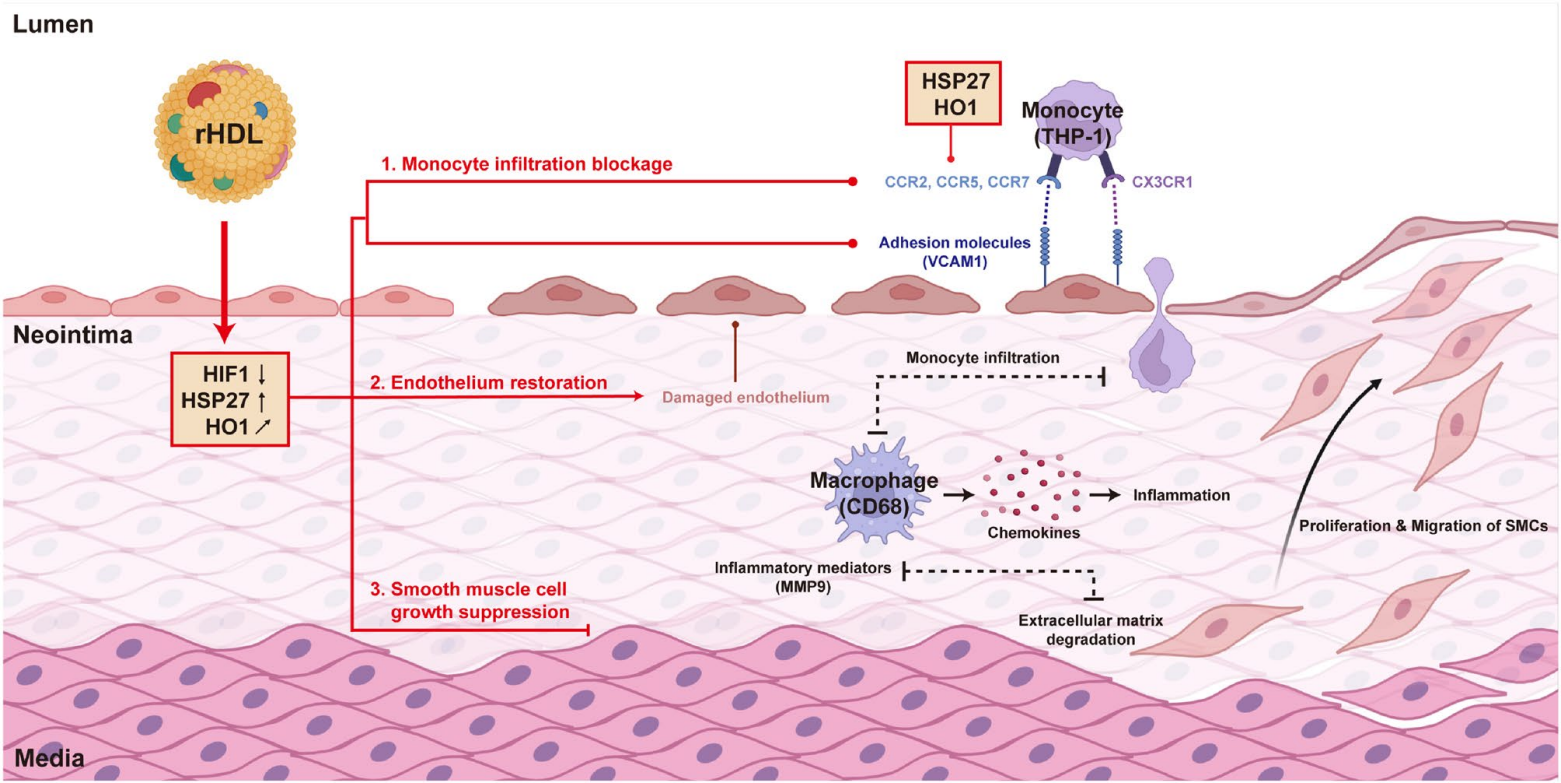
Summary of the effects of rHDL on anti-neointimal hyperplasia. Reconstituted high-density lipoprotein (rHDL) has demonstrated positive effects on the regulation of heat shock proteins (HSP27) and heme oxygenase-1 (HO1), leading to beneficial outcomes in anti-inflammatory reactions and cell proliferation. By inhibiting the expression of vascular cell adhesion molecules (VCAM1) on endothelial cells, rHDL effectively prevents the infiltration of immune cells such as neutrophils (CD18) and monocytes (CD68), thereby reducing inflammatory responses. Moreover, rHDL inhibits the differentiation of infiltrating cells into macrophages and decreases the secretion of cytokines like matrix metalloproteinase 9 (MMP9). In addition to its anti-inflammatory properties, rHDL plays a crucial role in regulating cell proliferation. It facilitates the repair of damaged and dysfunctional endothelial cells, promoting their re-endothelization. Furthermore, rHDL suppresses the excessive growth of vascular smooth muscle cells found in the media layer of blood vessels. Consequently, through its modulation of HSP27 and HO1 expression, rHDL supports cell survival by promoting endothelial recovery, suppressing smooth muscle cells, and restraining the infiltration of immune cells.

In summary, rHDL exerts distinct effects on endothelial cells, promoting their proliferation, while simultaneously suppressing the proliferation of SMCs. Furthermore, rHDL effectively mitigates hypoxic stress by reducing the expression of HIF1α and MMPs, ultimately leading to a reduction in neointimal hyperplasia following balloon injury in the carotid artery. As illustrated in Fig. 7, the infiltration of monocytes, which plays a crucial role in cell chemotaxis, adhesion, and egression at the site of injury, is partly regulated by chemokine receptors. The overexpression of HO1 and HSP27 in SMCs suppresses cell proliferation, thereby contributing to the inhibitory effect of rHDL on neointimal hyperplasia. Based on these findings, rHDL shows promise as a potential therapeutic option for patients undergoing balloon angioplasty and stenting, offering the potential to prevent and regress in-stent restenosis. Targeting the HO1 and/or HSP27 genes may provide novel therapeutic strategies in this context, although further research is needed to fully understand the underlying mechanism.

### Supplementary Information

Supplementary Information.

## Data Availability

The datasets used and/or analyzed during the current study available from the corresponding author on reasonable request.
