# Supplementary material for: Mechanistic insights into the anti-restenotic effects of HSP27 and HO1 modulated by reconstituted HDL on neointimal hyperplasia
Source: Sci Rep. 2023 Dec 12;13:22078. doi: 10.1038/s41598-023-49367-9 (PMC10716395; doi:10.1038/s41598-023-49367-9)
Supplement: Supplementary file 1 — Supplementary Information. [file 41598_2023_49367_MOESM1_ESM.pdf]

SUPPLEMENTARY INFORMATION

FOR

MECHANISTIC INSIGHTS INTO THE ANTI-RESTENOTIC EFFECTS OF HSP27 AND HO1 MODULATED BY RECONSTITUTED HDL ON NEOINTIMAL HYPERPLASIA

Ye Ji Kim[1,2,8+], Zinah Hilal Khaleel[1,2,8+], Myeongji Jin[1,2+], Jo Woon Yi Lee[3+], Seongchan Park[1,2], Seongmin Ga[1,2], Nam Hyeong Kim[1,2], Deok Hyang Sa[1,2], Eun Sung Kang[1,2], Seul Hee Han[3], Ji Yeun Lee[3], Hyo Jung Ku[3], Sang-Wook Kim[4], Ki Yong Kim[4], Jeong Euy Park[5,*], Yong Ho Kim[1,2,6,7,8,*], and Bok-Soo Lee[1,2,3,*]

[1]SKKU Advanced Institute of Nanotechnology (SAINT), Sungkyunkwan University, Suwon 16419, Republic of Korea

[2]Department of Nano Science and Technology, Sungkyunkwan University, Suwon 16419, Republic of Korea

[3]Division of Cardiology, Samsung Biomedical Research Institute, Sungkyunkwan University School of Medicine, Seoul, South Korea.

[4]Protein research Lab, CRC, GC Biopharma R&D center, Green Cross Co., Yongin 16924, Republic of Korea

[5]Division of Cardiology, Samsung Medical Center, School of Medicine, Sungkyunkwan University, Seoul 06351, Republic of Korea

[6]Department of Nano Engineering, Sungkyunkwan University, Suwon 16419, Republic of Korea

[7]Department of Biomedical Engineering, Sungkyunkwan University, Suwon 16419, Republic of Korea

[8]Center for Neuroscience Imaging Research (CNIR), Institute for Basic Science (IBS), Suwon 16419, Republic of Korea

[4]Present affiliation:

Sang-Wook Kim[4],

Samsung Bioepis PD team, 76 Songdogyoyuk-ro, Yeonsu-gu, Incheon, 21987, Republic of Korea

Ki Yong Kim[4],

Genexine BioResearch Institute, 172 Magocjungang-ro, BioInnovationPark, Bldg. Gangseo-gu, Seoul, 07789, Republic of Korea

This PDF file includes:

Supplementary Methods

Supplementary Tables 1 and 2

Supplementary Figures 1 to 8

SUPPLYMENTARY METHODS

Preparation of reconstituted HDL

In first step, 150 kg of precipitate IV of human plasma was washed with 3 volumes (w/v) of buffer (70 mM sodium citrate, 80 mM sodium phosphate, pH 4.0) for 2 hours and the precipitate was suspended in 4 volumes of 6 M urea for 2 hours at room temperature. The suspension was filtered and obtained a precipitate of 17-37% range by adding polyethylene glycol. The precipitate was suspended in 300 L of buffer (30 mM Tris-HCl, pH 8.0) and 600 L of ethanol. The suspension was filtered, the pH of the filtrate lowered to 6.0 and the resulting apoA-I precipitate was collected by further filtration. The precipitate was solubilized in 100 L of 4 M guanidine HCl and pasteurized for 10 hours at 60°C. The pH was then adjusted to 7.5 and applied to anion-exchange chromatography. The purified apoA-I was filtrated with 20 nm filter.

Subsequently, rHDL with a molar ratio of apoA-I to PC of 1:150 was prepared. The above apoA-I solution was mixed with lipid solution of 1.24 kg soybean PC and 715 g sodium cholate in 10 mM Tris-HCl, 10 mM NaCl, 1 mM EDTA, pH 7.5 for 12 hours at 5°C. After the mixture was diafiltered with at least 5 volumes of a 1% sucrose solution, sucrose was added to a final concentration of 10% and the concentration of the lipoprotein solution was adjusted to 20 mg/ml. After a final sterile filtration, the rHDL was filled in bottles of 1 g rHDL (protein weight) and lyophilized. The dry product was stored at -4°C in the dark until used. Before infusion, the lyophilized product was reconstituted with non-pyrogenic water for injection. The rHDL showed a disc-shaped, noncovalently associated particles resembling nascent HDL (Fig.1).

Confocal Microscopy analysis

All confocal image acquisition was done by either Leica SP8 confocal microscopy or confocal laser scanning microscopy 700 (CLSM 500) tools and image processing software packages used. Fluorescent labelling was observed with a confocal laser scanning microscope (CLSM700, Leica, Germany) equipped with a × 40 apochromatic oil immersion objective (NA: 1.2). Acquisitions were performed under exactly the same conditions. The excitation wavelength for Dapi/Hoechst 33342 was set at 405 nm and collected between 421 and 517 nm. The excitation wavelength for Alexa Fluor 488 was set at 488 nm and collected between 494 and 552 nm. The excitation wavelength for Alexa Fluor 568 was set at 578 nm and collected between 565 and 603 nm. The pinhole aperture was set at one Airy Unit (AU). Images were processed with the Image J® software version 2.1.0 (National Institutes of Health, USA).

SUPPLEMENTARY TABLES

Supplementary Table 1.

**Primer name**	**Sequence (5'-3')**
siHSP27-sense	GCUGCAAAAUCCGAUGAGAC=tt
siHSP27-antisense	GUCUCAUCGGAUUUUGCAGC=tt
siHO1-sense	CCUUGUACCAUAUCUAUAC=tt-
siHO1-antisense	GUAUAGAUAUGGUACAAGG=tt
Scrambled siRNA-sense	AUGAAACUGUUGUCAGCGCUG=tt
Scrambled siRNA-antisense	CAGCGCUGACAACAGUUUCAU=tt

Supplementary Table 2.

siRNA name	Sequence (5’-3’)
GAPDH-Fwd	GAGAAGGCTGGGGCTCATTT
GAPDH-Rev	AGTGATGGCATGGACTGTGG
HSP27-Fwd	ACGGTCAAGACCAAGGATG
HSP27-Rev	TTATTACTTGGCGGCAGTCT
HO1-Fwd	GTGCTCAAAAAGATTGCCCA
HO1-Rev	ATCTTGCACTTTGTTGCTGG
VCAM1-Fwd	TACCCATTTGACAGGCTGGA
VCAM1-Rev	TTGCATTTCCAGAAAGGTGC
CCR2-Fwd	CCACATCTCGGTTTATCAG
CCR2-Rev	CGTGGAAAATAAGGGCCACAG
CCR5-Fwd	GCAACATGCTGGTCATCCTC
CCR5-Rev	ACAGCCCTGTGCCTCTTCTT
CCR7-Fwd	CACAGTGCTCTCCATCCCAG
CCR7-Rev	CCGATGAAGGCGTACAAGAA
CX3CR1-Fwd	TCCGCAATGTGGAAACAAAT
CX3CR1-Fwd	ACTTCCATGCCTGCTCCTTT

SUPPLEMENTARY FIGURES

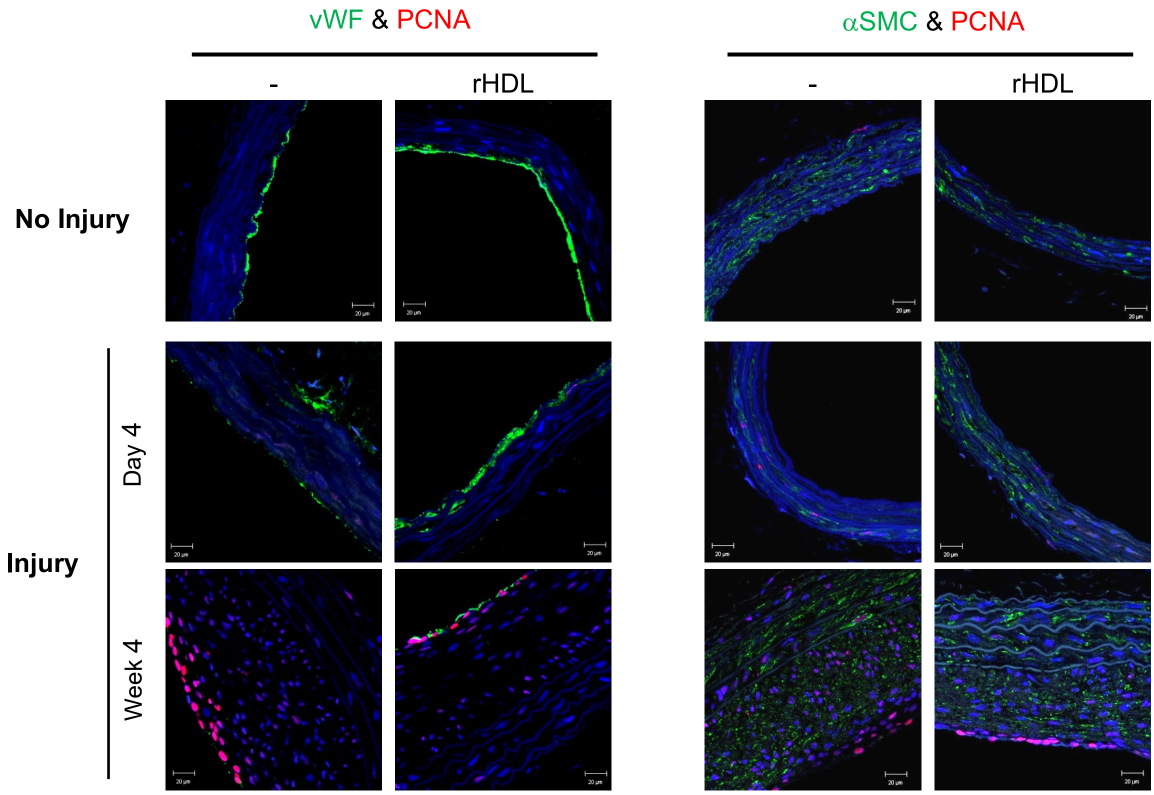

Supplementary Fig. 1. rHDL inhibit the proliferation of endothelial cells (ECs) and smooth muscle cells (SMCs) **in injured lesions**

Uncropped original confocal images of Fig. 2b are presented. To evaluate and compare the distribution and co-localization patterns of cell proliferation marker (PCNA) with endothelial cells or SMC, double staining was performed. The staining was conducted utilizing antibodies against vWF to label endothelial cells in green or antibody against SMC actin to label SMCs in green and PCNA to label nuclei in red. Then, tissue sections were stained with Hoechst33342 to identify nuclei, which resulted in a blue signal. These stained tissue sections were subsequently subjected to imaging using a confocal laser scanning microscope (CLSM700, Leica, Germany). The scale bar was set at 20 μm.

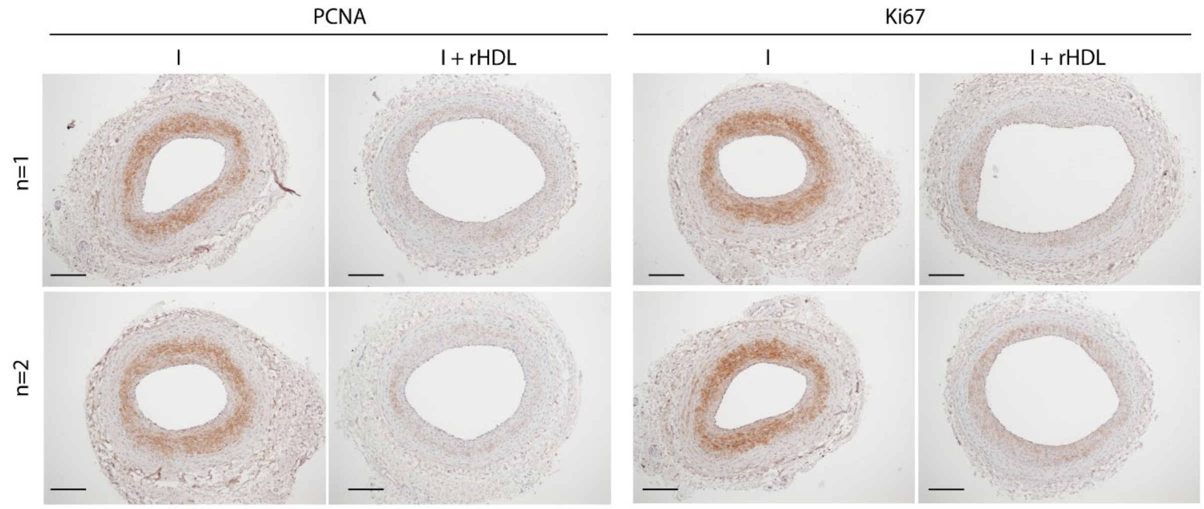

Supplementary Fig. 2. rHDL decreases cell proliferation in neointimal area

To assess cell proliferation in balloon injured carotid arteries at Week 4, tissue sections were subjected to staining using cell proliferation markers, PCNA or Ki-67. The sections were first deparaffinized and rehydrated, followed by a 30 min pretreatment with H_2O_2, as outlined in Methods section. Staining was carried out as per the specified procedure, The developed tissue sections by DAB were visualized under Eclipse 80i light microscope. Scale bar was set at 100 μm.

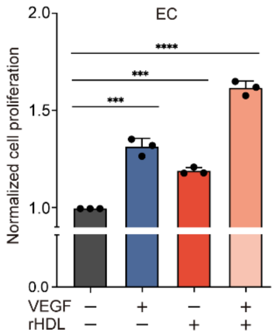

Supplementary Fig. 3. rHDL increases human umbilical vein endothelial cells (HUVEC) proliferation

Triplicated HUVECs were triplicated in culture and incubated for 48 hrs in the presence or absence of 100 ug/ml rHDL. To access cell proliferation, a Cell Counting Kit-8 (CCK-8) assay were performed by measuring the absorbance at 450 nm after 1 hr incubation. (***p<0.001, ****p<0.0001 verse control group, one-way ANOVA, n= 3)

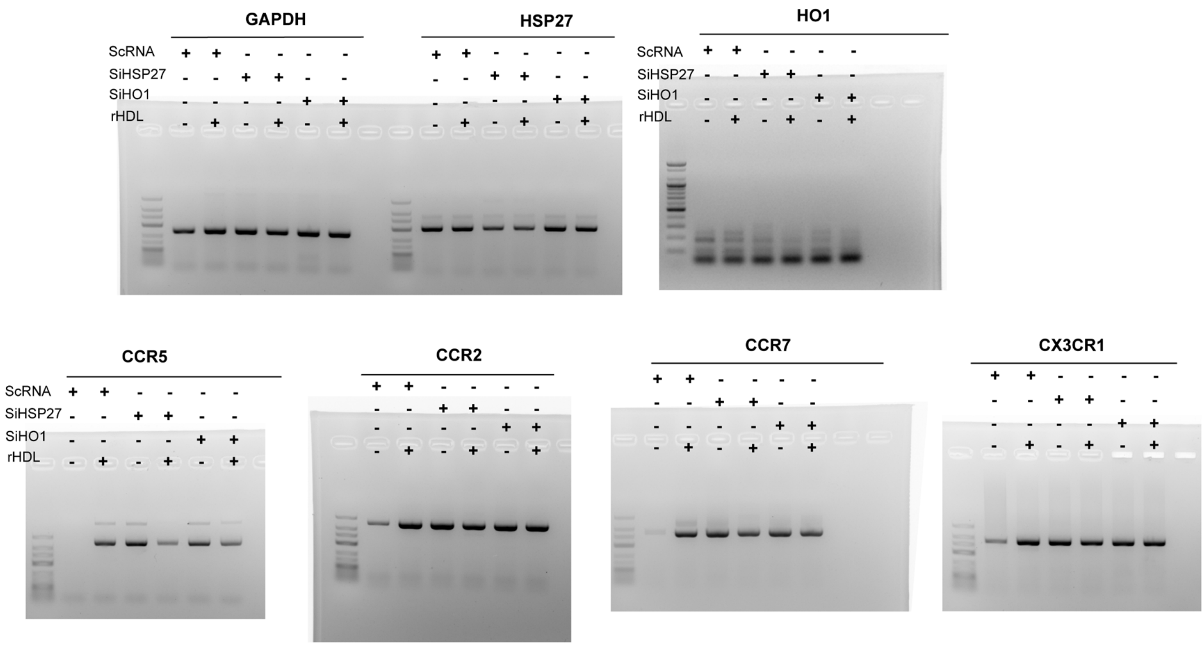

Supplementary Fig. 4. Gel-based RT-PCR analysis of chemokine receptors, CCR2, CCR5, CCR7, and CX3CR1 in THP1 cells.

A representative uncropped image of Fig. 4, that illustrates the impact of rHDLon chemokine receptors under conditions of HSP27 and HO1 knockdown. THP1 cells were transduced with siHSP27 or siHO1 targetting HSP27 or HO1 to specifically reduce the expression of these target genes. Subsequently, these cells were cultured in the presence or absence of 50 μg/ml rHDL for 30 hr. After isolation and quantification of total RNAs, cDNAs were synthesized using cDNA synthesis kit. The expression of CCRs was then assessed through RT-PCR to investigate the effect of rHDL under the aforementioned conditions of target knock-down.

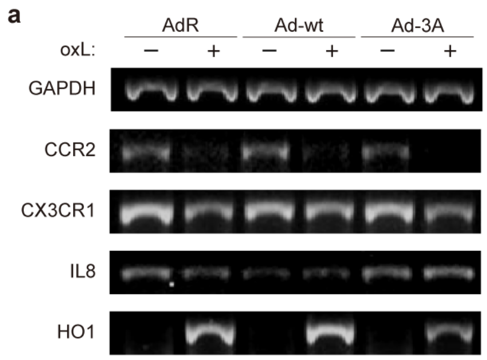

Supplementary Fig. 5. Effect of HSP27 phosphorylation status on CCRs and IL8 expression

a. Adenoviral constructs were prepared, including adenoviralvector alone (AdR), adenoviral vector with wild type HSP27 (Ad-wt), and adenoviral vector with 3 Ser residue to Ala in three well-known phosphorylation sites in HSP27 (Ad-3A). THP1 cells were infected with three viral constructs for 24 hr and treated with oxidized LDL (oxLDL) for 16 hr. Then, the expression of CCR2, CX3CR1, IL8 and HO1 was analyzed by RT-PCR.

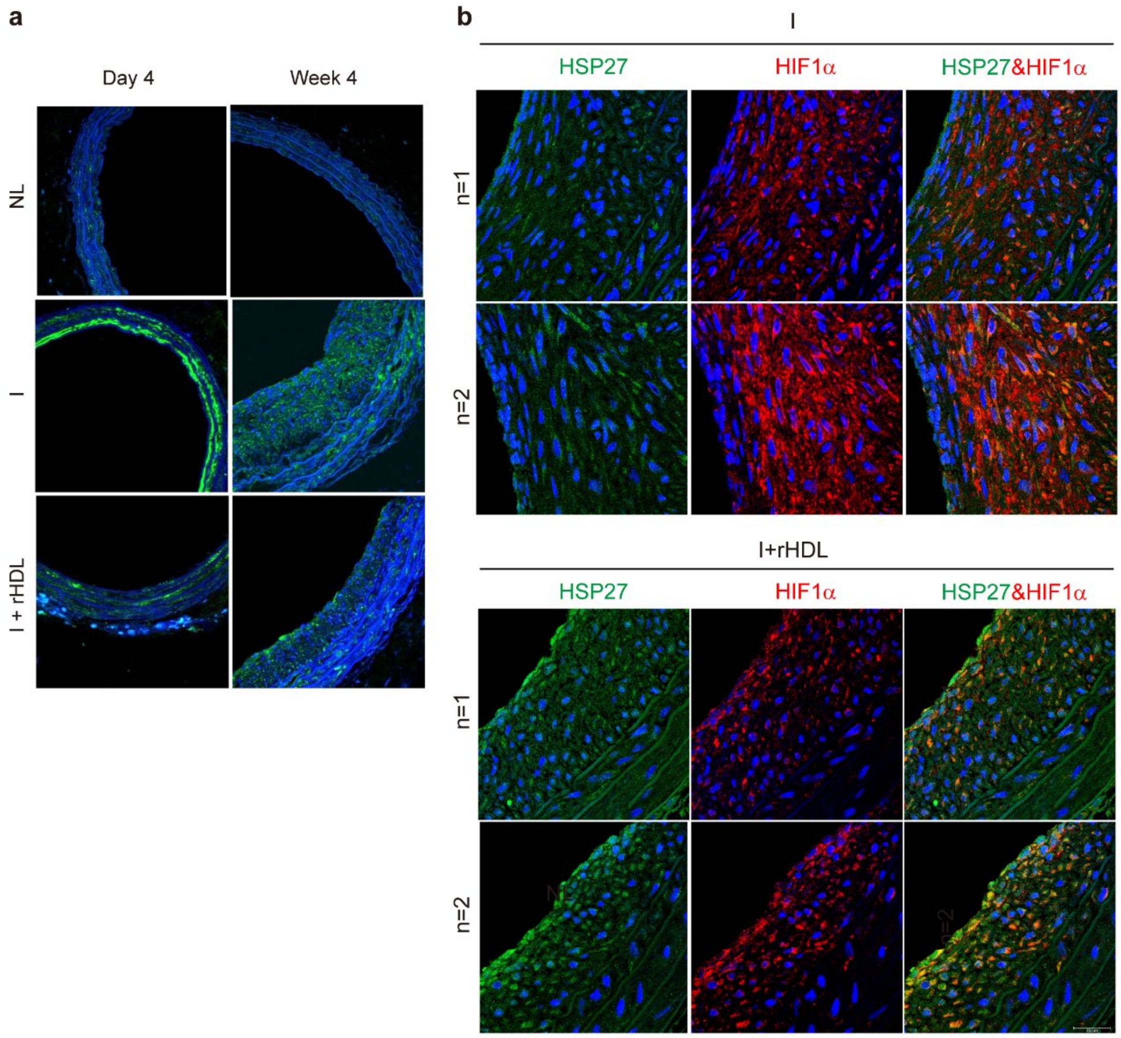

Supplementary Fig. 6. HIF1α expression in balloon injured rat carotid arteries

a. The sections of balloon-injured rat carotid arteries from normal group (NL), balloon injury group (I), and rHDL infused group (I + rHDL) at day 4 and week 4 after balloon injury were stained with anti-HIF1α antibody to observe hyperplasia affected by hypoxia. HIF1α were shown in green and nuclei were shown in blue by DAPI staining. **b.** The expression level of HSP27 (green) and co-localization signal with HIF1α (red) at week 4 were shown in two additional images. The images were acquired throughconfocal microscopy (CLSM700, Leica SP8).

a

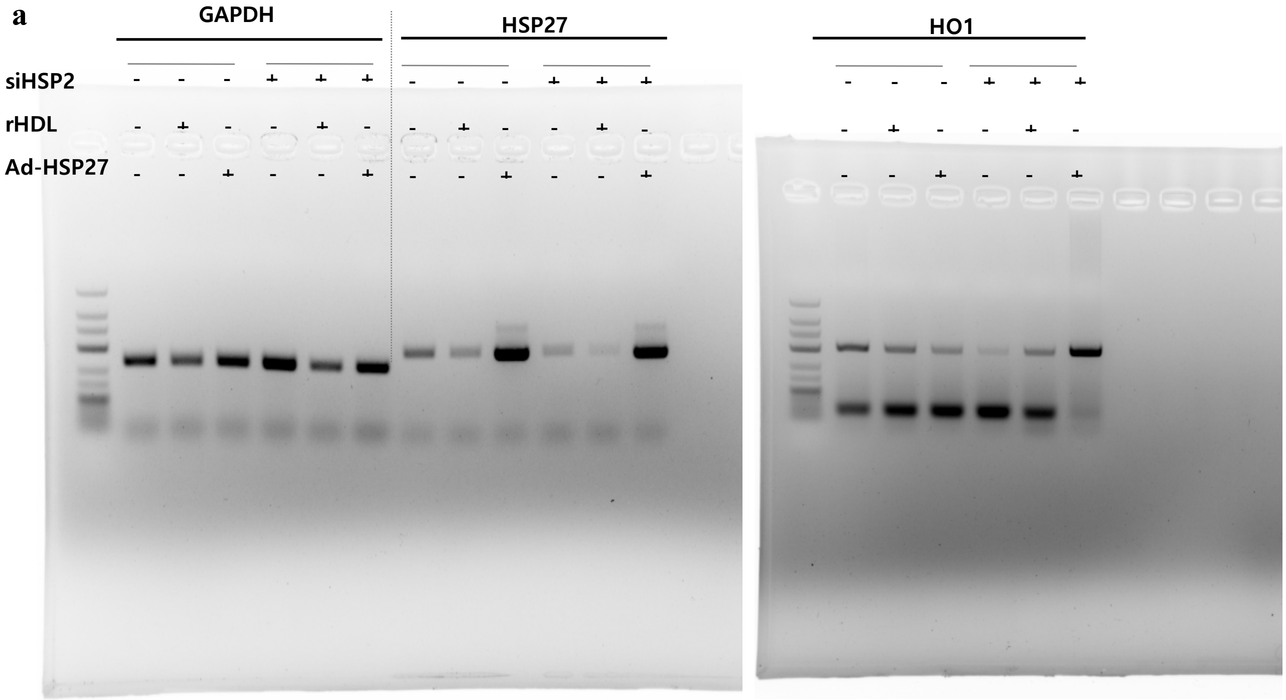

b

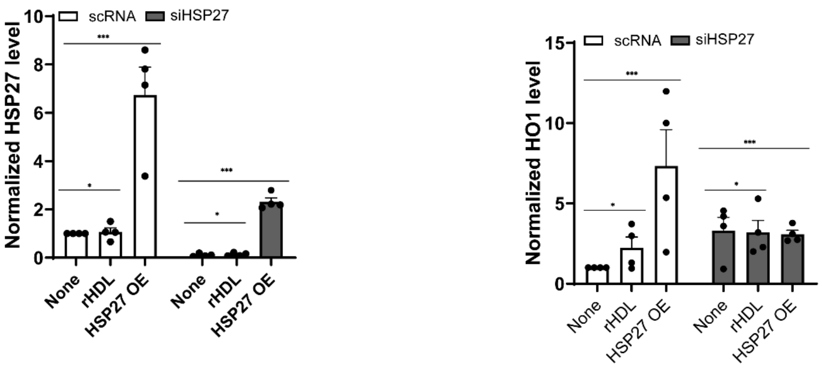

Supplementary Fig. 7. rHDL-mediated SMC proliferation via HSP27

A single representative, uncropped gel image from Figure 5f is presented. Loss or gain of function of HSP27 on SMC proliferation were examined after HSP27 knock-downed followed by with or without HSP27 overexpression by Ad-HSP27 infection in the presence or absence of 100 μg/ml rHDL. To interpret the exact role of HSP27 on SMC proliferation, the expression level of HSP27, HO1, and GAPDH in the experimental setting was confirmed by RT-PCR.

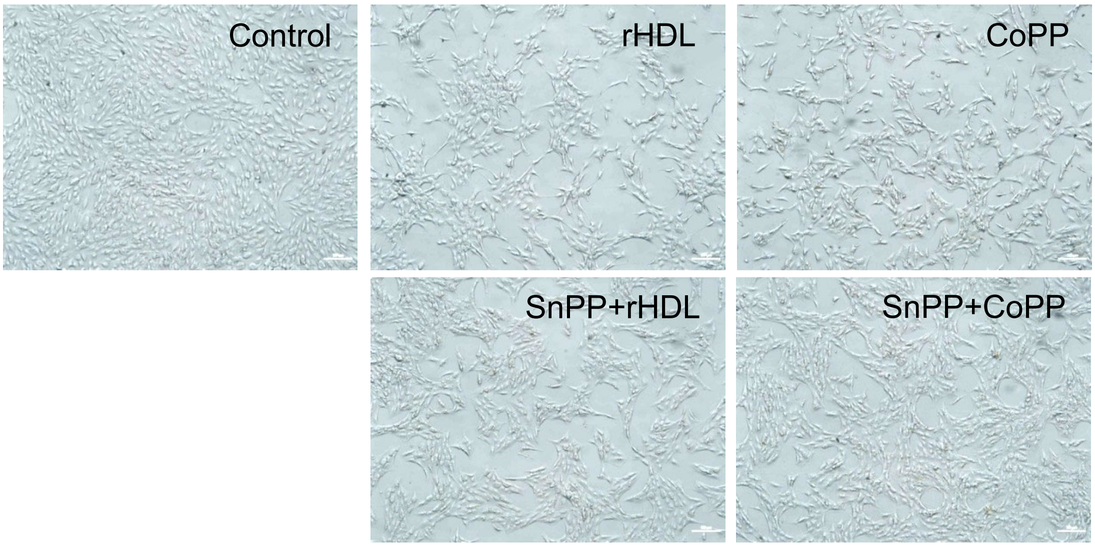

Supplementary Fig. 8. Inhibitory effect of HO1 on rHDL-mediated SMC proliferation *in vitro*

Uncropped images from Figure 6b are provided. SMCs were cultured for 48 hr in the presence or absence of rHDL or cobalt protoporphyrin (CoPP) with or without tin protoporphyrin (SnPP). After fixation, cells were visualized using light microscopy (Olympus, Magnification X100).
